# Clinical Characteristics and Outcomes of Children With Extracorporeal Membrane Oxygenation in a Developing Country: An 11-Year Single-Center Experience

**DOI:** 10.3389/fped.2021.753708

**Published:** 2021-11-16

**Authors:** Wirapatra Iamwat, Piya Samankatiwat, Rojjanee Lertbunrian, Nattachai Anantasit

**Affiliations:** ^1^Division of Pediatric Critical Care, Department of Pediatric, Faculty of Medicine, Ramathibodi Hospital, Mahidol University, Bangkok, Thailand; ^2^Division of Cardiothoracic Surgery, Department of Surgery, Faculty of Medicine, Ramathibodi Hospital, Mahidol University, Bangkok, Thailand

**Keywords:** extracorporeal membrane oxygenation, pediatric, risk factors, outcome, neonatal

## Abstract

**Introduction:** Extracorporeal Membrane Oxygenation (ECMO) is a lifesaving procedure for patients with refractory cardiac or respiratory failure. The indications for ECMO are growing, and it is increasingly being used to support cardiopulmonary failure in children. However, the risks and benefits of ECMO should be weighed before deploying it on the patients. The objectives of this study were to identify the mortality risk factors and to determine the ECMO outcomes.

**Methods:** The retrospective chart reviews were done for all patients aged 1 day−20 years old receiving ECMO between January 2010 and December 2020.

**Results:** Seventy patients were enrolled in the study. The median age was 31.3 months. The incidence of VA and VV ECMO was 85.7 and 14.3%, respectively. The most common indication for ECMO was the failure to wean off cardiopulmonary bypass after cardiac surgery. Pre-existing acute kidney injury (OR 4.23; 95% CI 1.34–13.32, *p* = 0.014) and delayed enteral feeding (OR 3.85, 95% CI 1.23–12.02, *p* = 0.020), and coagulopathy (OD 12.64; 95% CI 1.13–141.13, *p* = 0.039) were associated with the higher rate of mortality. The rates of ECMO survival and survival to discharge were 70 and 50%, respectively.

**Conclusion:** ECMO is the lifesaving tool for critically ill pediatric patients. Pre-existing acute kidney injury, delayed enteral feeding, and coagulopathy were the potential risk factors associated with poor outcomes in children receiving ECMO. However, ECMO setup can be done successfully in a developing country.

## Introduction

Extracorporeal Membrane Oxygenation (ECMO) is a life-saving procedure that is used to treat patients who have failed to respond to the conventional treatments for cardiac or respiratory failure (1–3). According to the Extracorporeal Life Support Organization (ELSO) report 2016, 24% of all ECMO implants were performed on children, and newborns accounted for 47% of the total cohort (4). Prior to ECMO initiation, the patient's risks and benefits should be outlined. Hemorrhagic and thrombotic complications are among the most common complications associated with ECMO. Nosocomial infections are also common in patients receiving ECMO and are associated with poor outcomes, especially in the neonatal population. Bartlett et al. performed the first neonatal ECMO in 1975, saving the life of a 1-day-old neonate with severe meconium aspiration syndrome (5). Following that, ECMO had been evolved in terms of equipment and management. Furthermore, this sophisticated technology had been successfully used to perform emergency cardiopulmonary resuscitation (E-CPR) (1, 6, 7). The Extracorporeal Life Support Organization (ELSO) gathered data and reported on the global growth, outcomes, and complications of the ECMO technology. In neonatal and pediatric patients, two primary ECMO circuits are used: venovenous (VV) and venoarterial (VA) ECMO. Vascular access is determined by the patient's condition and the surgeon's experience. While VA ECMO maintains both the hemodynamic and the respiratory function, VV ECMO only supports the respiratory function. ECMO is never recommended in the presence of lethal chromosomal or syndromic abnormalities, as well as severe irreversible brain or multiorgan failure.

The most common short-term complications in patients receiving ECMO were hemorrhagic and thrombotic events, cardiac depression, seizures, and acute kidney injury (AKI) (8–10). Long-term complications included hearing loss, delayed development, and decreased lung volume, particularly in patients with respiratory failure (10–13). Our hospital has been developing an ECMO program since 2003. Our center is the tertiary referral center in Bangkok, Thailand. Many patients have multiple conditions, including those who have undergone cardiac surgery, severe acute respiratory distress syndrome, solid organ transplantation, and heart transplantation. Each year, the number of pediatric patients receiving ECMO at our center increases. The objective of this study was to assess the outcomes of ECMO and to identify the risk factors for mortality in neonatal and pediatric patients receiving ECMO.

## Materials and Methods

This study was performed in a tertiary care referral center. We retrospectively analyzed the medical records of children aged 1 day−20 years who received ECMO from January 2010 to December 2020. This study was approved by the Institutional Review Board. Incomplete medical records were excluded. The demographic and baseline characteristics of the patients were collected, including their age, gender, body weight, immunological status, underlying disease, The Pediatric Risk of Mortality III, length of intensive care unit stays, and in-hospital mortality. Additionally, the characteristics of ECMO were reviewed, including indication, type of ECMO, duration of cannulation, and complications.

Initially the ECMO program was run by an individual attending cardiothoracic surgeon who reviewed the ELSO guideline and literature. Since 2015, our center has established an ECMO committee which comprised of pediatric intensivists, cardiothoracic surgeons, anesthesiologists, perfusionists, and certified ECMO nurses to assess the risks and benefits of ECMO in each patient. A cardiothoracic surgeon was initially ECMO program director with responsibility for overall operation. Two to three medical directors were responsible for locating an ECMO fund, establishing ECMO facilities and equipment, training in ECMO education, and preparing an annual ECMO summary. As the financial restrictions, the selected ECMO patients were required to undergo ECMO under the supervision of two of three ECMO committee members other than the patient's attending staff. These patients would be waived the ECMO expense from our center. Additionally, ECMO patients would have shortened the duration of the initial ECMO. As the lack of perfusionists, our center had only four to seven perfusionists who were responsible for cardiopulmonary bypass (CPB) and ECMO machine in both adults and children. Occasionally, we had three ECMO patients in the same period. There were insufficient perfusionists to cover all ECMO patients during 24-h on-call period. We had initiated a local basic and advanced ECMO course to train ICU nurses to become certified ECMO nurses capable of caring for the ECMO machine in place of the perfusionist. We had annually local ECMO meeting and workshop to keep updated ECMO knowledge and skills to the certified ECMO physicians and nurses. The indications of ECMO were patients who had the potentially reversible causes of severe cardiopulmonary failure such as pediatric acute respiratory distress syndrome with oxygen index > 30 for 6 h or >40 for 2 h, acute fulminant myocarditis, failure to weaning from CPB, progressive ventricular failure, or severe pulmonary hypertensive crisis. The location of ECMO cannulation was specified as an operating room or pediatric intensive care unit (PICU). Cannulation configurations were classified as central (sternotomy) for patients undergoing cardiopulmonary surgery or peripheral vessel for patients undergoing medical cardiopulmonary failure. We had the initial standing order in place for the ECMO-selected patients. The ECMO circuits were primed with packed red blood cells if the patient's weight was <10 kg. Heparin 100 unit/kg was given as a bolus infusion with a goal of an activated clotting time of 300 s. The initial pump flow rate was 20–30 mL/kg/min and increased gradually to the target flow of 2.4–3 L/m^2^/min. Typically, the initial sweep gas flow rate was equal to the blood flow rate (1:1), and the sweep flow was adjusted according to the PaCO_2_ level of each patient. Adjustment of pump flow was used to ensure adequate systemic perfusion (central venous oxygen saturation > 70%, normal arterial lactate, and adequate mean arterial blood pressure). The anticoagulant was using unfractionated heparin with monitoring *via* activated clotting time, activated partial thromboplastin time, and antiXa level. We had the multidisciplinary team including ICU physicians, ICU nurses, certified ECMO nurses, perfusionists, pediatric nutritionists, pediatric hematologists, clinical pharmacists. Weaning from ECMO was initiated when the following criteria were met: improvement in clinical course, recovery of end-organ function, and stable respiratory and hemodynamic status. Weaning from the VA ECMO was accomplished by decreasing the ECMO flow to 30 mL/kg/min, bridging ECMO for 1 h, and monitored the hemodynamic status and central venous oxygen saturation. If successful bridging was achieved, the decannulation of ECMO was performed. The protocol for weaning from VV ECMO was to decrease the FiO_2_ in the ECMO to 0.21 and weaned the sweep gas to zero. We then monitored the oxygen saturation and the arterial blood gases. If the oxygen saturation was >92% and the arterial blood gases were within the normal range, the VV ECMO could be then decannulated.

## Definitions

Successful ECMO weaning was defined as the patients who survived for more than 72 h after successful cessation of ECMO support (14).

Hospital survival was defined as the patients who were successfully weaned off from ECMO and continued to survived until hospital discharge.

Early enteral feeding was defined as enteral feeding within 48 h after ECMO cannulation, whereas delayed enteral feeding was defined as enteral feeding after 48 h of ECMO cannulation.

The definition of acute kidney injury (AKI) was defined according to the Kidney Disease Improving Global Outcome (KDIGO) (15). The pre-existing AKI was defined as patients who had developed AKI within 48 h prior to initiate ECMO or after 48 h of ECMO initiation.

## Statistical Methods

Data were analyzed using PASW Statistics, version 21 (SPSS Inc., Chicago, IL, USA). Descriptive statistics were presented as frequency, distribution, and percentage. Differences in the frequencies of discrete variables were tested using Pearson's Chi-square test or Fisher's exact test. Logistic regression was used to measure the association between the clinical variable and hospital mortality. Risk factors determined to be clinically significant a priori on the bivariable analyses were identified as candidate variables for the multivariable model. A log-rank test was used to analyze the survival of patients who received ECMO. A *p*-value of <0.05 was considered statistically significant.

## Results

**Figure 1:**
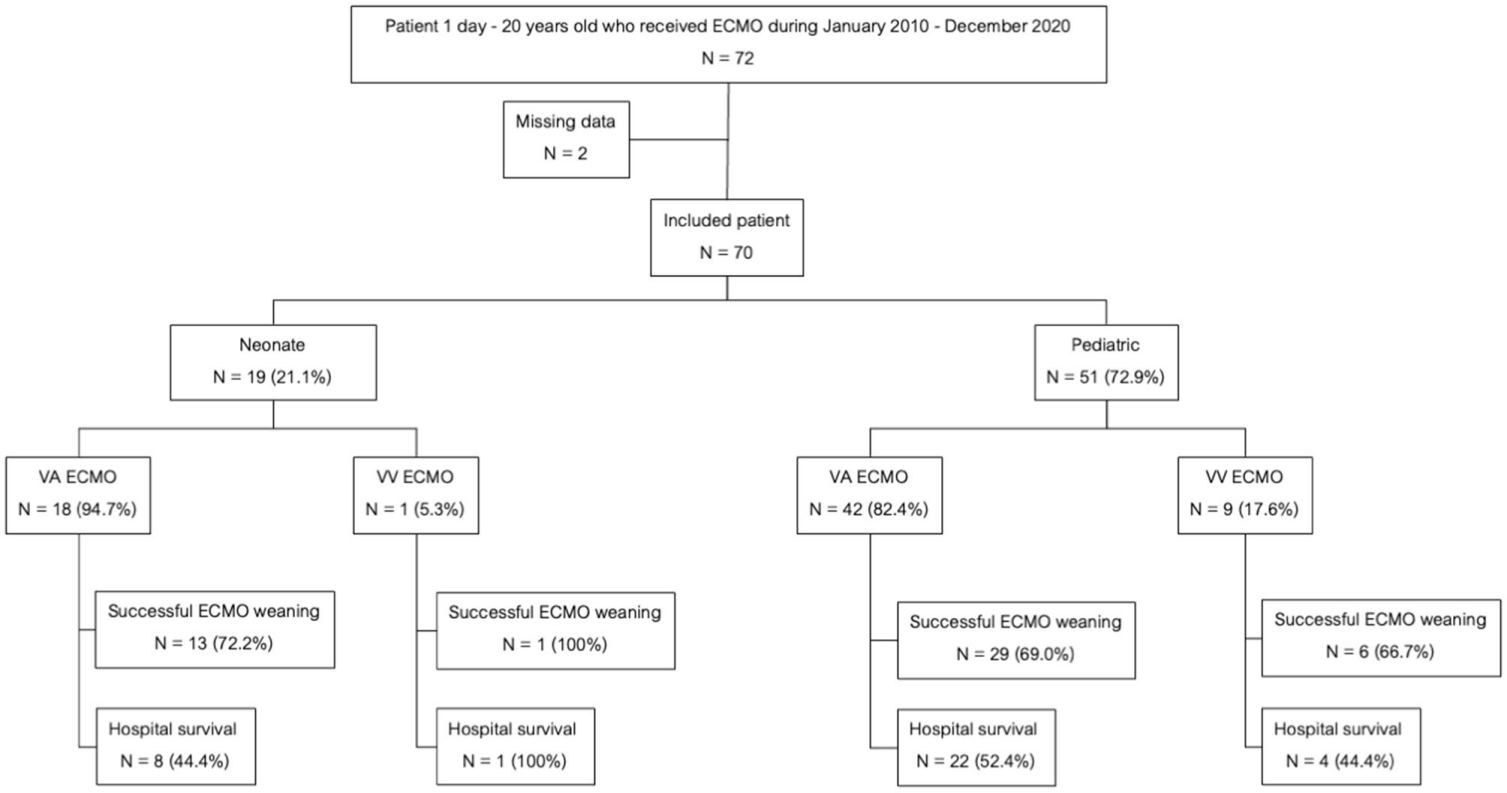
Flow chart of pediatric ECMO patients in this study.

A total of 70 children underwent ECMO support during the study period. Fifty-one patients (72.8%) were among the pediatric group while the remaining 19 (27.2%) were in the neonatal group. There were 60 (85.7%) patients required VA ECMO and 10 patients (14.3%) required VV ECMO. The flow chart of all patients receiving ECMO support in this study was shown in Figure 1. There were 4 and 66 patients who received ECMO support during 2010–2015 and 2015–2020, respectively. The mortality rate was not significantly different between two periods (75 vs. 48.5%, *p* = 0.326). The baseline characteristics were shown in Table 1. Central cannulation was the most frequently used site for cannulation in neonatal and children. Central cannulation was performed in 71% of cases. The mean age was 31.3 (0.9–46.4) months, and 58.6% of participants were male. Forty-nine (70%) patients were successfully weaned from ECMO. Overall hospital mortality was 50%. Among the pediatric group, the mortality rate was 25 of 51(52.6%) patients while the neonatal group suffered a mortality rate of 10 of 19 (49%) patients. The mortality rate of VA ECMO and VV ECMO were 50 and 50%, respectively. The most common comorbidity was congenital heart disease. The three major indications for ECMO support the failure to wean off cardiopulmonary bypass (CPB) (34.3%), ventricular failure (34.3%), and pulmonary hypertensive crisis (17.1%). Twenty-nine patients (41.1%) had pre-existing AKI stage 2 or above. Two patients who required ECMO for bridging to heart transplantation and both of them survived.

**Table 1:** Baseline characteristics.

	**Total (*N* = 70)**	**Survivors(*N* = 35)**	**Non-survivors (*N* = 35)**	***p*- value**
Median age (months; IQR)	31.3 (0.9–46.4)	13.4 (1–52.1)	7.4 (0.7–45)	0.443
Gender: Male, *n* (%)	41 (58.6)	21 (60.0)	20 (57.1)	0.808
Median body weight (kg; IQR)	8.5 (3.3–15.2)	8.5 (3.8–15.0)	7.4 (3.2–15)	0.750
Underlying disease, *n* (%)				
Heart disease	51 (72.9)	25 (71.4)	26 (74.3)	0.764
Healthy	10 (14.3)	6 (17.1)	4 (11.4)	
Other	9 (12.9)	4 (11.4)	5 (14.3)	
Median PRISM III score (IQR)	15.5 (13–19)	16 (13–18)	14 (14–19)	0.522
Indications for ECMO, *n* (%)				
Failure to weaning from CPB	24 (34.3)	10 (28.6)	14 (40.0	0.461
Ventricular failure	24 (34.3)	15 (42.9)	9 (25.7)	
Pulmonary hypertensive crisis	12 (17.1)	5 (14.3)	7 (20.0)	
Severe ARDS	9 (12.9)	4 (11.4)	5 (14.3)	
In-hospital cardiac arrest	1 (1.4)	1 (2.9)	0	
ECMO year 2015–2020	66 (94.3)	34 (97.1)	32 (91.4)	0.364
ECMO type, *n* (%)				
Venoarterial	60 (85.7)	30 (85.7)	30 (85.7)	1.000
Venovenous	10 (14.3)	5 (14.3)	5 (14.3)	
Delayed enteral feeding, *n* (%)	33 (47.1)	11 (31.4)	22 (62.9)	0.008^*^
Pre-existing AKI, *n* (%)	29 (41.4)	9 (25.7)	20 (57.1)	0.008^*^

*PRISM III, The Pediatric Risk of Mortality III; CPB, cardiopulmonary bypass; ECMO, extracorporeal membrane oxygenation; ARDS, acute respiratory distress syndrome; AKI, acute kidney injury*.

*Median (interquartile range)*,

* *p-value < 0.05*.

The median duration of ECMO support was 8.3 days (range 3 h−27 days). The median length of stay in ICU was 29.8 days (range 3–134 days). The most frequently encountered complications were hematological (28.6%) and neurological in nature (28.6%). Table 2 summarized the outcomes and associated complications. The delayed enteral feeding was found to be higher among the non-survivors than survivors (62.9 vs. 31.4%, *p* = 0.008). Coagulopathy was found to be more prevalent in the non-survivors than the survivors (20 vs. 2.9%, *p* = 0.024). Only those who died in the hospital had disseminated intravascular coagulation (DIC). There were 5 patients with thrombocytopenia, 2 patients with heparin-induced thrombocytopenia (HIT) and only 1 patient with heparin resistance. Multiple logistic regression analysis to adjust for the clinical variables significantly associated with hospital mortality (age, PRISM III, delay enteral feeding, pre-existing AKI, and coagulopathy) confirmed that delayed enteral feeding [Odds ratio (OR) 3.85; 95% confidence interval (CI) 1.23–12.02; *p* = 0.020], pre-existing AKI [OR 4.23; 95% CI 1.34–13.32, *p* = 0.014], and coagulopathy [OD 12.64; 95% CI 1.13–141.13, *p* = 0.039] were potential risk factors for increasing mortality (Table 3).

**Table 2:** Outcomes and complications of ECMO.

	**Total (*N* = 70)**	**Survivors(*N* = 35)**	**Non-survivors (*N* = 35)**	***p-*value**
Duration of ECMO support (days, IQR)	6.5 (4–12)	6 (4–8)	8 (4–13)	0.069
Length of stay in ICU (days, IQR)	24 (15–36)	23 (15–36)	24 (14–40)	0.784
Length of stay in hospital (days, IQR)	30 (18–35)	37 (25–61)	24 (14–40)	0.395
Upper gastrointestinal bleeding, *n* (%)	6 (8.6)	1 (2.9)	5 (14.3)	0.088
Acute kidney injury, *n* (%)	10 (14.3)	3 (8.6)	7 (20.0)	0.172
Continuous renal replacement therapy, *n* (%)	4 (5.7)	1 (2.9)	3 (8.5)	0.303
**Immediate complications**, ***n*** **(%)**				
Surgical site bleeding	7 (10.0)	2 (5.7)	5 (14.3)	0.232
Pneumothorax	6 (8.6)	2 (5.7)	4 (11.4)	0.415
Hemopericardium	3 (4.3)	1 (2.9)	2 (5.7)	0.555
**Infectious complications**, ***n*** **(%)**				
CRBSI	5 (7.1)	2 (5.7)	3 (8.6)	0.643
VAP	8 (11.4)	3 (8.6)	5 (14.3)	0.452
**Neurological complications**, ***n*** **(%)**				
Seizure	10 (14.3)	5 (14.3)	5 (14.3)	1.000
Cerebral hemorrhage	7 (10.0)	4 (11.4)	3 (8.6)	0.690
Stroke	1 (1.4)	1 (2.9)	0	0.314
ECMO mechanical complications, *n* (%)	5 (7.1)	3 (8.6)	2 (5.7)	0.643
Coagulopathy, *n* (%)	8 (11.4)	1 (2.9)	7 (20.0)	0.024^*^

*ICU, intensive care unit; CRBSI, catheter-related blood stream infection; VAP, ventilator associated pneumonia*.

*Median (interquartile range)*,

* *p-value < 0.05*.

**Table 3:** Logistic regression analysis for associated risk factors with mortality.

**Variables**	**Univariate analysis Odd ratios (95%CI)**	**Multivariate analysisOdd ratios (95%CI)**	***p*-value**
Age	1.005 (0.994–1.016)	1.003 (0.989–1.018)	0.663
PRISM III	0.932 (0.841–1.032)	0.904 (0.784–1.042)	0.904
Delayed enteral feeding	3.692 (1.372–9.933)^*^	3.848 (1.232–12.021)^*^	0.020
Pre-exiting AKI	3.852 (1.401–10.590)^*^	4.227 (1.341–13.324)^*^	0.014
Coagulopathy	8.500 (0.986–73.276)	12.644 (1.133–141.127)^*^	0.039

*PRISM III, The Pediatric Risk of Mortality III; AKI, acute kidney injury*.

* *p-value < 0.05*.

**Figure 2:**
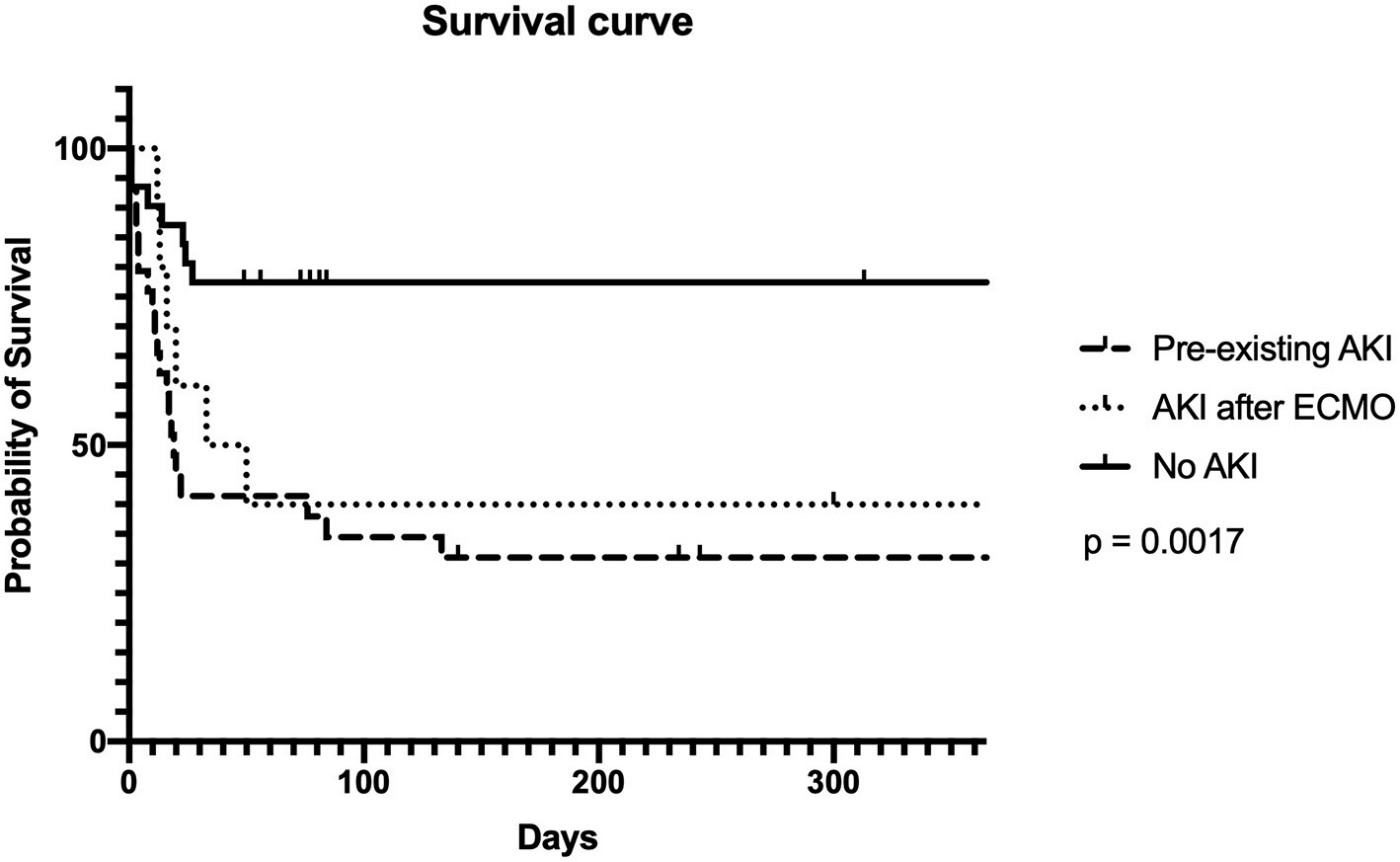
Survival curve.

Fifty percent of the neonatal and pediatric patients on ECMO support survived until discharge. The leading cause of in-hospital death was septicemia (54.3%). Other causes of death included heart failure (28.6%), brain death (5.7%), and renal failure (1.4%). The overall 1-year survival rate of our patients was 47.8%. Survival rates were 31.0, 30, and 73.3%, respectively, in the pre-existing AKI, AKI after ECMO, and the non-AKI group (*p* = 0.050) (Figure 2).

## Discussion

In this study, the distribution of age group among patients requiring ECMO varied similarly to the previously reported studies by ELSO, 85.7% in pediatric age group and 14.3% in neonates. This study found that the neonates with congenital heart disease had a higher rate of requiring VA ECMO implantation than the general population, which was consistent with the previous research (13). The most common indication for ECMO within this cohort was the failure to wean off cardiopulmonary bypass following cardiac surgery. The number of patients who received ECMO support climbed rapidly after the ECMO committee established. The mortality rate was slightly decreased but no significant difference in two period due to the very low number of ECMO patients in pre-ECMO committee program period. The ECMO survival rate and survival rate to discharge were 70 and 50%, respectively, which were comparable to the previous studies (12, 13, 16, 17). This study found the ECMO survival rate to discharge in VA and VV were 50%, while the ELSO registry summarized that the mortality rate of VA ECMO was 51.9% and VV ECMO was 61.8% in Asia pacific registration (18). Our ECMO program used unfractionated heparin as the routine anticoagulant. Our study found ECMO patients had HIT 2.8% and heparin resistance 1.4%. An overall incidence of immune-mediated HIT with unfractionated heparin was 2.6% by a meta-analysis (19). Bivalirudin is an alternative anticoagulant in ECMO for patients with HIT or heparin resistance (20). To our knowledge, this study was the first study to report the clinical characteristics, risk factors, and outcomes in pediatric patients with ECMO in Thailand.

The incidences of AKI in neonatal and pediatric ECMO patients were estimated to be between 50 and 62% in a multicenter retrospective observational cohort study (21). Additionally, patients with AKI were shown to require a longer duration of ECMO support, longer ventilator days, and had a higher mortality rate (17, 21, 22). A recent meta-analysis study in 3,523 pediatric patients on ECMO demonstrated that AKI was significantly associated with reduced survival outcome (23). AKI was the most frequently encountered complication in this study and was associated with a significant increase in mortality rate, particularly in those with pre-existing AKI. Additionally, we also found that the non-AKI group had a higher 1-year survival rate than the AKI group (31 vs. 73%, *p* = 0.050).

Providing optimal nutrition to critically ill children is also an important aspect of a favorable outcome. There were few nutritional support studies in pediatric patients who received ECMO even though they were nutritionally vulnerable (24, 25). A prior retrospective study showed that early enteral feeding was associated with a lower hospital mortality rate (25). Our study found that late enteral feeding after 48 h was associated with an increased risk of mortality.

Septicemia was the leading cause of death in our patients. The most prevalent pathogen was bacteria, particularly those with extensive drug resistance, which was consistent with other studies (26, 27).

Our study had several strengths. We reported the first clinical characteristics and outcomes of pediatric ECMO in Thailand. This study demonstrated a successful ECMO program that developing countries might use to drive the establishment of their own programs and improve patient outcomes. This program utilized an established ECMO committee to select ECMO patients, create certified ECMO nurses as ECMO specialists, establish ECMO training and continue annual ECMO training.

This study had some limitations. First, this was a retrospective study with some variables having missing data. Second, because this study was conducted in a single center with a small sample size, it cannot be generalized. Our center, on the other hand, was a large referral center that may represent the region. Finally, the federal government imposes spending limits on ECMO patients. ECMO may be initiated late in the course of some patients' illnesses. However, our center utilized an ECMO committee to determine which patients would receive ECMO, which likely shortened the time required to initiate ECMO. Additionally, this financial crisis may be comparable to that of other developing countries. Additional collaborative and prospective research are required to validate risk factors and outcomes in developing countries.

## Conclusion

ECMO is the lifesaving treatment for rescuing neonatal and pediatric patients with refractory cardiopulmonary failure. Pre-existing acute kidney injury and delayed enteral feeding were associated with an increased mortality rate. Nosocomial infection was the leading cause of death in hospitals. An ECMO program could be done successfully with a multidisciplinary team in developing country.

## Data Availability Statement

The original contributions presented in the study are included in the article/supplementary material, further inquiries can be directed to the corresponding author/s.

## Ethics Statement

The studies involving human participants were reviewed and approved by Human Research Ethics Committee, Faculty of Medicine Ramathibodi Hospital, Mahidol University. Written informed consent from the participants' legal guardian/next of kin was not required to participate in this study in accordance with the national legislation and the institutional requirements.

## Author Contributions

WI and NA contributed to design of the study, data collection, data analysis, and manuscript drafting. PS and RL contributed to design of the study. NA critically revised it for important intellectual content. All authors gave final approval of the version to be published.

## Conflict of Interest

The authors declare that the research was conducted in the absence of any commercial or financial relationships that could be construed as a potential conflict of interest.

## Publisher's Note

All claims expressed in this article are solely those of the authors and do not necessarily represent those of their affiliated organizations, or those of the publisher, the editors and the reviewers. Any product that may be evaluated in this article, or claim that may be made by its manufacturer, is not guaranteed or endorsed by the publisher.
